# Expectation violation and attention to pain jointly modulate neural gain in somatosensory cortex

**DOI:** 10.1016/j.neuroimage.2017.03.041

**Published:** 2017-06

**Authors:** Francesca Fardo, Ryszard Auksztulewicz, Micah Allen, Martin J. Dietz, Andreas Roepstorff, Karl J. Friston

**Affiliations:** a Danish Pain Centre, Department of Clinical Medicine, Aarhus University, 8000 Aarhus, Denmark; b Interacting Minds Centre, Aarhus University, 8000 Aarhus, Denmark; c Institute of Cognitive Neuroscience, University College London, London WC1N 3AR, United Kingdom; d Oxford Centre for Human Brain Activity, University of Oxford, Oxford OX3 7JX, United Kingdom; e Wellcome Trust Centre for Neuroimaging, University College London, London WC1N 3BG, United Kingdom; f Center for Functionally Integrative Neuroscience, Aarhus University, 8000 Aarhus, Denmark

## Abstract

The neural processing and experience of pain are influenced by both expectations and attention. For example, the amplitude of event-related pain responses is enhanced by both novel and unexpected pain, and by moving the focus of attention towards a painful stimulus. Under predictive coding, this congruence can be explained by appeal to a precision-weighting mechanism, which mediates bottom-up and top-down attentional processes by modulating the influence of feedforward and feedback signals throughout the cortical hierarchy. The influence of expectation and attention on pain processing can be mapped onto changes in effective connectivity between or within specific neuronal populations, using a canonical microcircuit (CMC) model of hierarchical processing. We thus implemented a CMC within dynamic causal modelling for magnetoencephalography in human subjects, to investigate how expectation violation and attention to pain modulate intrinsic (within-source) and extrinsic (between-source) connectivity in the somatosensory hierarchy. This enabled us to establish whether both expectancy and attentional processes are mediated by a similar precision-encoding mechanism within a network of somatosensory, frontal and parietal sources. We found that both unexpected and attended pain modulated the gain of superficial pyramidal cells in primary and secondary somatosensory cortex. This modulation occurred in the context of increased lateralized recurrent connectivity between somatosensory and fronto-parietal sources, driven by unexpected painful occurrences. Finally, the strength of effective connectivity parameters in S1, S2 and IFG predicted individual differences in subjective pain modulation ratings. Our findings suggest that neuromodulatory gain control in the somatosensory hierarchy underlies the influence of both expectation violation and attention on cortical processing and pain perception.

## Highlights

• Expectation and attention influence how pain is processed and perceived.
• Pain-related signals are processed throughout a hierarchy of somatosensory and fronto-parietal regions.
• Expectation violation and attention to pain modulate intrinsic and extrinsic connectivity in the somatosensory hierarchy.
• Both expectation violation and attention change the precision of pain-related signals in S1 and S2.
• Intrinsic and extrinsic connectivity parameters predicted subjective pain modulation ratings.

## Introduction

Expectation and attention both exert a strong influence on pain perception (e.g., Wiech et al., 2008) and the magnitude of pain-related neural responses (e.g., Dowman, 2007; Legrain et al., 2009b). Specifically, expectation violation and top-down attention have a similar effect on pain processing, as both unexpected and attended pain typically increase the amplitude of event-related potentials. Although the underlying neural mechanism of this common effect is unknown, one potential explanation lies in the theory of hierarchical predictive coding (Friston, 2005, Friston, 2008). This theory suggests that top-down and bottom-up signals are integrated by precision-dependent processes, where the precision or confidence afforded to ascending prediction error signals is encoded by postsynaptic gain (i.e., cortical gain control or excitation-inhibition balance; Feldman and Friston, 2010). Here, we tested the hypothesis that both expectation violation and attention effects on pain processing are mediated by a precision-weighting mechanism, using a canonical microcircuit model (CMC; Bastos et al., 2012) of cortical dynamics for magnetoencephalography (MEG).

The violation of sensory expectations is a salient event, which typically elicits increased neural activity, irrespective of the deviant stimulus feature or sensory domain (Mouraux et al., 2011). In electrophysiological studies, expectation violation has been extensively investigated with respect to the mismatch negativity (e.g., Näätänen et al., 2012), a well-characterized difference in neural response elicited by a novel stimulus embedded within a structured stream of repeated, identical stimuli. Although most frequently studied in the auditory domain (for reviews, see Näätänen et al., 2011, Näätänen et al., 2012), cortical mismatch responses have been observed for all sensory modalities, including somatosensation (e.g., Kekoni et al., 1997; Akatsuka et al., 2007b; Ostwald et al., 2012; Allen et al., 2016) and nociception (Hu et al., 2013a; Zhao et al., 2014; also see Legrain et al., 2002, Legrain et al., 2005).

Similar to expectation violation, the magnitude of pain-related potentials is also modulated by attention, irrespective of the somatosensory (Miltner et al., 1989, Yamasaki et al., 2000, Van der Lubbe et al., 2012) or nociceptive modality (Siedenberg and Treede, 1996, Legrain et al., 2002, Lorenz and Garcia-Larrea, 2003). Generally, attention enhances the sensitivity of neuronal populations encoding attended sensory inputs, while inhibiting neurons responding to irrelevant inputs (Desimone and Duncan, 1995). As in the case of mismatch responses, attention has been shown to enhance neuronal responses for a variety of perceptual modalities (Woldorff et al., 1993, Hillyard and Anllo-Vento, 1998, Eimer and Forster, 2003). This common effect on event-related potentials might suggest a similar neural mechanism underlying the facilitative influence of both expectation violation and attention on pain processing. Specifically, these effects can be reconciled in terms of bottom-up and top-down attentional selection of nociceptive inputs (Legrain et al., 2009a), mediated by changes in connectivity in somatosensory and fronto-parietal networks (Corbetta and Shulman, 2002). Under predictive coding, top-down (prior) and bottom-up (sensory) signals are integrated in proportion to their precision (i.e., inverse variance or uncertainty). Computationally, this corresponds to a weighting of prediction errors by their precision (Friston, 2009), and has been previously operationalized in terms of changes in post-synaptic neuromodulatory gain (Feldman and Friston, 2010). For instance, precision-weighting in the context of top-down visuo-spatial attention has been linked to the gain of superficial pyramidal cells encoding prediction errors, in a way that is consistent with biased competition (Brown and Friston, 2013).

To test the hypothesis that expectation violation and attention to pain are both mediated by a precision-weighting mechanism, we used dynamic causal modelling (DCM) of MEG responses to painful stimuli in a location-based roving oddball paradigm. First, we established the hierarchy of somatosensory and fronto-parietal regions underlying location-specific pain mismatch responses. Then, we used DCM to investigate how expectation violation and attention modulated the gain of superficial pyramidal cells, modelled by intrinsic self-connections, and the extrinsic connectivity between different neuronal populations in the somatosensory hierarchy. Our results establish that enhanced neural activity driven by expectation violation and attention to pain was similarly explained by increased precision-weighting or gain in superficial pyramidal cells in somatosensory cortex, while expectation violation also increased recurrent connectivity with fronto-parietal sources.

## Materials and methods

### Participants

26 healthy volunteers were recruited from Aarhus University and the local community. All participants were right-handed (Edinburgh Handedness Inventory; 93.81%±1.20) and had normal or corrected-to-normal vision. No participants reported a history of pain disorders, neurological or psychiatric illness, or use of analgesics. All participants received a reimbursement of 500 DKK for participation and gave their informed consent before participation. Data from two participants were not included in any analyses due to technical failures during data collection. Two further participants were excluded from statistical analysis on account of excessive MEG artifacts. The final sample included 22 participants (12 females; mean age=23 years; range=20–29 years). The study was approved by the Ethical Committee of the Central Region Denmark and conducted in accordance with the Declaration of Helsinki.

### Stimuli, task and procedure

Painful stimuli were delivered using two intra-epidermal electrodes (Inui et al., 2002, Inui and Kakigi, 2012), via two Digitimer DS7A stimulators (Digitimer, Hertfordshire, UK). One concentric bipolar needle electrode was placed on the dorsum of each hand, over the radial nerve. Each stimulus consisted of two rapid square-wave pulses of 50 μs duration; with an inter-pulse interval of 5 ms. Stimulus intensity was calibrated for each participant (and for each hand) to induce a painful percept of 5 on a visuo-analog scale from 0 to 10. The calibration was obtained via a staircase procedure, based on the 3 up 1 down rule, 6 reversals and decreasing intensity steps of 1, 0.5, 0.2 and 0.1 mA. Participants rated the intensity of each stimulus on a horizontal visual-analogue scale (VAS; range=0–10, where 0 equals ‘‘no pain sensation’’, 1 “just noticeable pain” and 10 ‘‘worst imaginable pain”). A single intensity, corresponding to the arithmetic mean of the intensity levels identified as “5” in the two hands, was used in the experimental task.

**Fig. 1:**
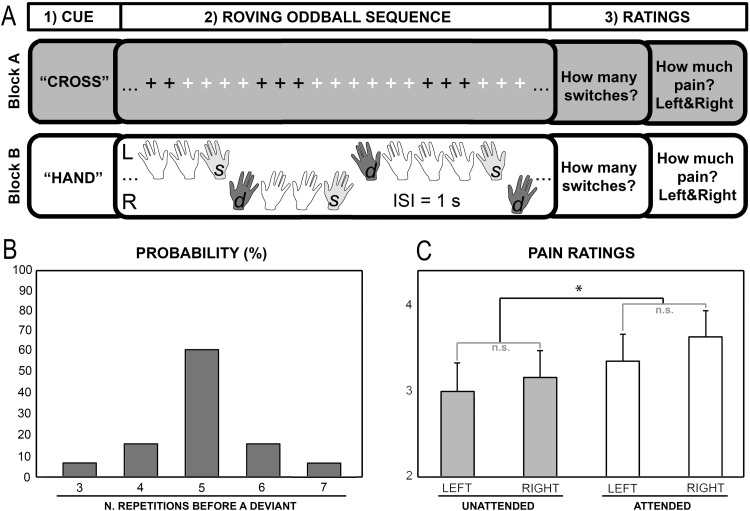
**A) Experimental task.** Top-down attentional set was manipulated at the block level by presenting a verbal instruction to attend away from pain and towards the visual stimulation on the screen (i.e., cue=“CROSS”; unattended pain) or attend to the painful stimuli perceived on the dorsum of the hands (i.e., cue=“HAND”; attended pain). In each block, a total of 25 trains of painful stimuli were delivered to the dorsum of one hand at the time, using an oddball roving sequence. Each train included 3–7 stimulus repetitions at a constant inter-stimulus interval of 1 s. Each painful stimulus consisted of two rapidly square-wave pulses of 50 μs duration, with an inter-pulse interval of 5 ms. The same stimulus intensity was used for both left and right stimuli. We considered a deviant (d) the first stimulus in each train (i.e., change in stimulus location). To match the number of trials of deviant and standard stimuli, we only modeled the last repetition before a change as a standard (s). While painful stimuli were delivered, a fixation cross on the screen changed in color from black to white or vice versa every 2–5 s. The visual change never occurred at the same time as a change in the painful stimulus location. When instructed to pay attention to the visual stimulation, participants had to silently count the number of time the cross changed in color from white to black or vice versa (Block A). Instead, when instructed to pay attention to the painful stimuli, participants had to silently count the number of times the stimulation switched from the left to the right hand or vice versa (Block B). Block order was counterbalanced across participants. At the end of each block, participants were required to report the number of switches, as well as to rate the average pain intensity experienced for each hand. **B)** The probability of repetitions between 3–7 times was 5%, 15%, 60%, 15%, 5%, respectively. **C)** Mean and standard error of pain ratings for left and right hand and attended (white) and unattended (grey) pain, separately. Participants reported less intense pain when the painful somatosensory stimuli were unattended.

The experimental task consisted of a within-block manipulation of sensory expectation using a roving oddball sequence (Garrido et al., 2008, Ostwald et al., 2012, Allen et al., 2016) and a between-block manipulation of top-down attention using instructions to attend to – or away from – pain before each block (Fig. 1A). Within blocks (n=8), sensory expectations were implicitly established by the roving oddball sequence, given the higher probability of stimulus repetitions in the same location (i.e., pain on the same hand) with respect to a change in spatial location (i.e., pain on the other hand). After a minimum of 3 and a maximum of 7 painful stimulus repetitions on the same hand, the location of the stimulation switched to the dorsum of the other hand repeatedly, throughout the duration of each block (Fig. 1B).

The switch (i.e., deviant stimulus) commonly elicits an automatic shift of attention towards the novel spatial location, particularly when the unexpected sensory event is inherently salient or behaviorally relevant. To establish temporal predictability of the stimulus sequence, the inter-stimulus interval was held constant at 1 s. To define the event-related responses of interest, the first painful stimuli of each roving oddball sequence (i.e., pain from the unexpected spatial location) driving a bottom-up shift of attention were defined as deviants. For example, left deviants corresponded to unexpected painful stimuli delivered on the left hand, after several stimulus repetitions on the right hand. Conversely, the last painful stimuli of each repeated train (i.e., pain from the expected spatial location) were referred to as standards. For example, left standards consisted of expected painful stimuli on the left hand, after several stimulus repetitions on the same location. To ensure a balanced analysis, we modeled only one repetition in each oddball sequence as standard (i.e., the last repetition before a change in location). The roving oddball sequence ensured that left and right deviant and standard events had identical stimulus features, but differed only with respect to expectancy. Similar roving sequences have been extensively used to study mismatch responses in different sensory domains, including the somatosensory (Ostwald et al., 2012, Allen et al., 2016) and nociceptive systems (Hu et al., 2013a; Zhao et al., 2014).

Between blocks, top-down attention was manipulated via verbal cues informing the participants to either attend towards pain (i.e., attended pain) or away from pain (i.e., unattended pain). Each block began with the visual presentation of a word, either “HAND” (n=4 blocks) or “CROSS” (n=4 blocks), informing the participant of the upcoming block condition. When the cue “HAND” was presented, participants were asked to pay attention to the painful stimuli delivered on the dorsum of the hands and silently count the number of times the stimulation switched from one hand to the other (i.e., pain attentional set), while ignoring visual stimuli (i.e., cross changing in color). Conversely, when the cue “CROSS” was presented, participants were asked to ignore the painful stimuli, pay attention to the fixation cross at the center of a screen and to silently count the number of times the color of the cross changed from white to black or vice versa (i.e., visual attentional set). The active counting task was chosen to control for attentional effort throughout the roving sequences; the cross fluctuated in all attention conditions. As deviants (i.e. stimulus changes) were unpredictable, this manipulation requires participants to maintain similar attentional effort to deviants and standards within the attended sensory modality.

Overall, participants received a total of 1000 painful stimuli, including 200 deviants equally distributed across the four conditions defined by laterality (i.e., left, right) and attention (i.e., attention to pain vs. away from pain). The probability of repetitions between 3 and 7 times was 5%, 15%, 60%, 15%, 5%, respectively (Fig. 1B). Blocks contained on average 24 painful deviants (min=17, max=31), and 37 color changes (min=28, max=49). Following each block, participants were asked to report the exact number of events counted during the previous block, either changes in spatial location or changes in the color of the fixation cross, using a numerical rating scale from 10 to 60. Further, they were asked to rate the average pain felt on the left and right hands on a visual analogue scale from 0 to 10 (where 0 equals “no pain sensation,” 1 “just noticeable pain” and 10 ‘‘worst imaginable pain”). After each rating session, 5 s rest intervals separated contiguous blocks. The blocks were presented with two possible pseudo-randomized sequences to counterbalance order effects: ABBABAAB or BAABABBA. Before the beginning of the experiment, participants completed a brief training session comprising two blocks, one for each attention condition. All participants reported that the two blocks were sufficient to understand the task. The PsychoPy software package v1.76.00 (Peirce, 2007, Peirce, 2009) was used for instructions, stimulation and presentation of VASs.

### Behavioral and subjective statistical analysis

Detection accuracy of changes in pain spatial location and in the color of the fixation cross were compared using paired t-tests. Further, the subjective ratings of the perceived pain intensity were analyzed using two-way repeated-measures ANOVAs, with the within-subject factor “attention” (2 levels: attended and unattended pain) and “laterality” (2 levels: left and right hand). Statistical significance was set at p<.05, effect sizes were calculated using the partial η_2_, and the Tukey HSD test was applied for post-hoc comparisons.

### MEG acquisition and preprocessing

MEG data was acquired using an Elekta Neuromag TRIUX MEG system with 204 planar gradiometers and 102 magnetometers. Blinks and eye movements were monitored using vertical and horizontal bipolar surface electrodes. The data were digitized with a sampling frequency of 1 kHz, with analog filtering of 0.1–330 Hz. A continuous measure of the head position with respect to the sensors was obtained using four head-position indicator coils attached to the scalp. Further, three fiducial markers (i.e., nasion, left and right pre-auricular points) and around 100 scalp points were digitized to define a MEG coordinate frame.

The raw MEG signal was maxfiltered (MaxFilter 2.2.15 software; Elekta Neuromag) to (1) remove externally generated noise using the temporal extension of the signal source separation (tSSS) algorithm (Taulu and Simola, 2006); (2) detect bad channels automatically; (3) correct for head movements within session; and (4) correct for head positions across participants. Further, preprocessing and statistical analysis of MEG data were implemented using SPM12 (Statistical Parametric Mapping 12, http://www.fil.ion.ucl.ac.uk/spm). The raw (maxfiltered) data were epoched into 700 ms stimulus time-locked epochs (−100/+600 ms), baseline corrected using the average pre-stimulus activity at −100/−15 ms and downsampled to 300 Hz. Robust averaging was applied for artifact removal (Wager et al., 2005), as this method down-weights the contribution of extreme values that do not occur at the same time points across trials (i.e., outliers).

Averaging was computed separately for each condition, leading to 8 average waveforms corresponding to unattended left deviant (uLD), unattended left standard (uLS), unattended right deviant (uRD), unattended right standard (uRS), attended left deviant (aLD), attended left standard (aLS), attended right deviant (aRD), and attended right standard (aRS). These eight averages correspond to the cells of our 2×2×2 factorial design with three factors (deviant vs. standard, right vs. left, and attended vs. unattended) After combining the planar gradiometers, we converted the ERF time-series for each condition into three-dimensional scalp maps over two-dimensional sensor-space (x, y) and time (z) (Kilner and Friston, 2010, Litvak et al., 2011). For each participant, each time point of the averaged conditions was transformed into a two dimensional 64×64 pixel scalp map using linear interpolation and concatenated over the interval from 20 to 600 ms. The resulting 3D scalp map volumes (i.e., 8 images for each participant) were smoothed with a low-pass kernel (6 mm×6 mm×6 ms full-width at half maximum, FWHM) and entered into a general linear model (GLM) for statistical parametric mapping. The time interval of interest for the statistical analysis did not include the baseline period, which by definition cannot differ across conditions and subjects.

### Statistical parametric mapping – sensor space analysis

We conducted a general linear model (GLM) mass-univariate SPM analysis on the combined planar gradiometers. The maximal activity observed at the scalp level is approximately located superior to the source, providing an easier interpretation of the scalp maps (Hämäläinen et al., 1993). In a group-level 2×2×2 repeated measures ANOVA, we modeled the experimental conditions (8 levels; uLS, uLD, uRS, uRD, aLS, aLD, aRS, aRD) and the factor subject (22 levels), in order to assess the main effect of laterality, attention, and expectation violation, as well as the attention by expectation violation and laterality by expectation violation interactions. Inferences were corrected for multiple comparisons, across sensors and time points, using Gaussian random field theory to control the cluster-wise error rate (Kilner and Friston, 2010, Litvak et al., 2011). The threshold for significant results was set at an uncorrected peak-level selection threshold at *p*<.005 and at *p*<.05, family-wise error (FWE) corrected at the cluster level.

### Dynamic causal modeling – source space analysis

Using DCM and Bayesian model comparison, we first specified the network architecture of a somatosensory processing hierarchy, in terms of extrinsic connections between somatosensory and fronto-parietal sources identified in an auxiliary source-localization analysis. Having identified the optimal network architecture, we then asked how expectation violation and attention modulated between-region (extrinsic) and within-region (intrinsic) effective connectivity within this somatosensory hierarchy. We used Bayesian Model Averaging to summarize the posterior probability of our grand-average model parameters. Finally, we inverted the winning model separately for each subject to test whether individual differences in subjective pain modulation correlated with intrinsic and extrinsic connectivity parameters.

#### Source localization

**Fig. 2:**
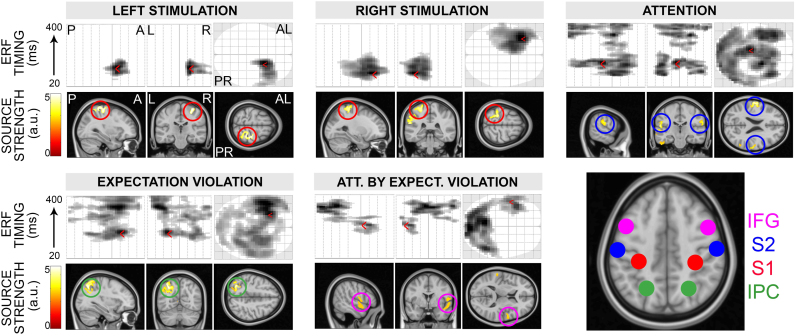
**ERF and source reconstruction results.** Summary of laterality (separately for left and right), attention, expectation violation main effects, and attention by expectation violation interactions. In each panel, the first row depicts the timing and topography of event-related field effects at the scalp level. The left figure represents the posterior-anterior displacement of the effect as a function of time (y axis, from 20 to 400 ms). The red arrow indicates the ERF maximal peak; e.g., in central-anterior locations, at around 80 ms (left stimulation) and 50 ms (right stimulation) and in a central location at around 100 ms (attention). The central figure represents the left-right displacement as a function of time (y axis, from 20 to 400 ms). Again, the red arrow indicates the ERF maximal peak; e.g., in the right hemisphere (left stimulation), left hemisphere (right stimulation) or close to the midline (attention). Finally, the right figure depicts the topography of the ERF effect at the peak time point; e.g., over anterior right sensors (left stimulation), anterior left sensors (right stimulation), widespread across posterior and anterior sensors (attention). The second row illustrates the topography of reconstructed sources. The analysis identified four bilateral sources (S1, S2, IFG, and IPC). The specific locations of left and right S1 sources (MNI coordinates: left [−26, −36, 58]; right [32, −40, 64]) were derived by comparing right vs. left and left vs. right stimulation (i.e., laterality main effect). We established bilateral S2 coordinates (MNI coordinates: left [−62, 14, 20]; right [62, 24, 26]) by comparing attended vs. unattended pain, regardless the stimulation side. Finally, we identified left inferior frontal and right inferior parietal regions (MNI coordinates: left IFG [−54, 8, 16]; right IPC [36, −66, 40]) in the expectation violation main effect, as well as right inferior frontal and left inferior parietal regions (MNI coordinates: right IFG [54, 0, 10]; left IPC [−32, −64, 46]) in the attention by expectation violation interaction. The identified sources were then entered into a dynamic causal modelling specifying alternative connectivity architectures. (For interpretation of the references to color in this figure legend, the reader is referred to the web version of this article.)

To establish the optimal network architecture underlying the evoked responses, we first performed source localization on the observed signals using a minimum-norm procedure (Hämäläinen and Ilmoniemi, 1994, Litvak et al., 2011). The time window considered was between 20 and 400 ms. The time interval from 0 to 20 ms was excluded as it contained artefactual activity due to the electrical stimulation. We identified eight cortical sources (Fig. 2) consisting of bilateral primary and secondary somatosensory cortices (S1 and S2), inferior parietal cortex (IPC) and inferior frontal gyrus (IFG). The specific locations of left and right S1 sources (MNI coordinates: left [−26, −36, 58]; right [32, −40, 64]) were derived by comparing right vs. left and left vs. right stimulation (i.e., laterality main effect). We established bilateral S2 coordinates (MNI coordinates: left [−62, 14, 20]; right [62, 24, 26]) by comparing attended vs. unattended pain, regardless the stimulation side. Finally, we identified left inferior frontal and right inferior parietal regions (MNI coordinates: left IFG [−54, 8, 16]; right IPC [36, −66, 40]) in the expectation violation main effect, as well as right inferior frontal and left inferior parietal regions (MNI coordinates: right IFG [54, 0, 10]; left IPC [−32, −64, 46]) in the attention by expectation violation interaction.

#### Predictive coding, DCM, and CMC

Predictive coding entails a neurobiological implementation of the inferential processes supporting perception, based on recurrent message-passing in cortical hierarchies (Friston, 2010). The core notion of predictive coding lies in the integration of top-down predictions (i.e., descending signals) and bottom-up prediction errors (i.e., ascending signals). Prediction errors – discrepancies based on current predictions and the inputs from hierarchically lower regions or (at the peripheral level) from the sensorium – are passed to higher-order regions in a feedforward fashion, to update high level representations. Conversely, predictions descend to lower-order regions to suppress, or explain away, prediction errors. The influence of prediction errors at each level of the hierarchy is weighted by their relative precision or reliability. For example, precise prior beliefs at higher levels of the hierarchy can override sensory impressions or conversely, reliable (precise) sensory evidence can override prior beliefs regardless of how unexpected they are. The constitutive elements of perceptual inference; namely, prediction, prediction error, and precision weighting can be mapped onto particular neurobiological mechanisms; in particular, various subpopulations in canonical microcircuits and their postsynaptic gain on excitability. Clearly, to interpret neuronal responses and connectivity in terms of predictive coding one has to adopt a model of canonical microcircuity.

Accordingly, the CMC model postulates four neural populations associated with distinct ascending and descending connectivity streams that are integrated within each cortical column (Bastos et al., 2012). Spiny stellate and deep pyramidal cells are modelled as receiving ascending or bottom-up inputs, while superficial pyramidal cells and inhibitory interneurons are modelled as receiving descending or top-down inputs. In terms of predictive coding, superficial pyramidal cells signal prediction errors to higher-order regions, while deep pyramidal cells signal predictions to lower-order regions. Crucially, the intrinsic excitability (or gain) of superficial pyramidal cells can now be interpreted as encoding the precision of prediction errors^1^ (Feldman and Friston, 2010). This interpretation appears to have a degree of validity in relation to attentional gain and enjoys the support of several empirical studies (e.g., Fogelson et al., 2014; Pinotsis et al., 2014; Auksztulewicz and Friston, 2015; Vossel et al., 2015). The implementation of the CMC model in DCM therefore enabled us to test the directionality and modulation of the message passing in terms of extrinsic connectivity (i.e., between-region passing of prediction errors and prediction), as well as intrinsic connectivity (i.e., self-inhibition or gain modulation representing the precision of prediction errors). Note that CMC/DCM does not provide direct evidence of how distinct neurons respond to sensory inputs. Instead, it represents an estimate based on a neuronal mass model (see below, *Canonical Microcircuit Model*).

#### Dynamic causal modelling

**Fig. 3:**
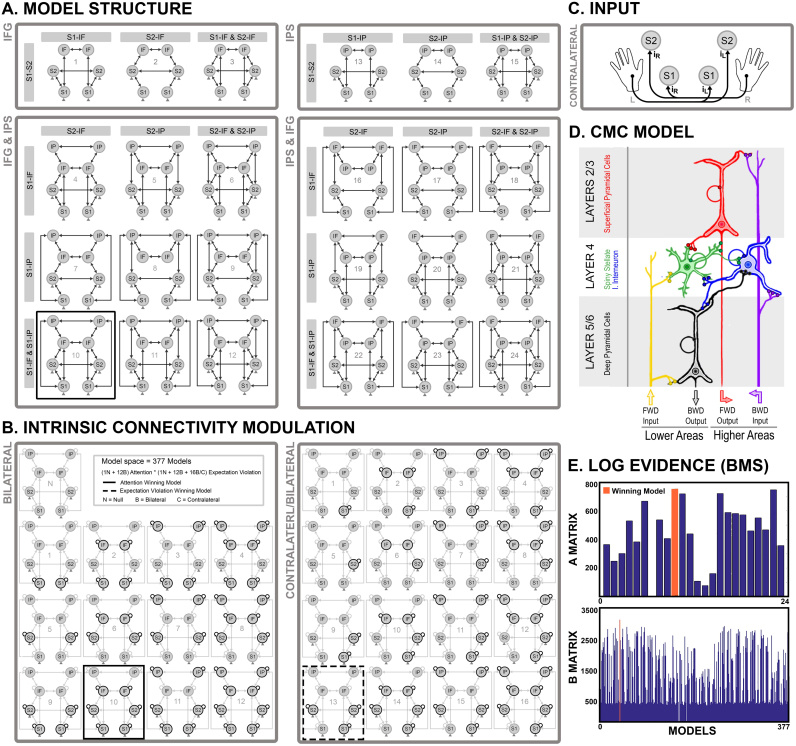
**A)** Architectures of 24 alternative models of somatosensory and fronto-parietal regions, fitted to grand-averaged ERF data. The models differed with respect to the inclusion of a frontal (IFG) and/or parietal (IPC) node, as well as the hierarchical architecture and connections between somatosensory and fronto-parietal areas. **B)** Contextual modulations of intrinsic connectivity by attention and expectation violation were optimized with respect to 1 null model, 12 bilateral alternative models (attention and expectation violation) and 16 contralateral somatosensory models (expectation violation). In the figure, the contralateral models are shown for left expectation violation. Right expectation violation models were identical but with left lateralization of somatosensory regions. **C)** Both contralateral S1 and S2 were specified as cortical targets of thalamic input. **D)** All DCMs were tested using a canonical microcircuit model. Each source was thus modeled as compromising 4 neuronal populations (superficial and deep pyramidal cells, spiny stellate and inhibitory interneurons). **E)** The winning model structure (M10), identified using fixed-effect Bayesian model selection, included all bilateral regions, with IPC at the highest hierarchical level, as well as connections between S1 to both fronto-parietal nodes and connections between S2 and the frontal node. **E)** The winning model of connectivity modulation, identified using fixed-effect Bayesian model selection, included changes in gain in bilateral primary, secondary somatosensory cortex, as well as and IFG by attention (M10). Further, the winning model revealed changes in contralateral primary and bilateral secondary somatosensory cortex by expectation violation (M13).

The cortical regions identified in the source localization were entered into a set of dynamic causal models that embodied alternative connectivity architectures. The connectivity structure was initially optimized by considering 24 alternative networks of somatosensory and fronto-parietal regions (Step 1; Fig. 3A). We then optimized the intrinsic and extrinsic connectivity changes associated with our experimental manipulation of expectation violation and attention, by inverting and averaging models from a factorial model space (Step 2; Fig. 3B). In both DCM analyses, the peristimulus time window was 20–400 ms. Sources were modeled as equivalent current dipoles and corresponded to cortical patches of 16 mm radius, centered on the locations above. Both contralateral S1 and S2 were specified as cortical targets of thalamic input (i.e., left-hand inputs to right S1 and S2; right-hand inputs to left S1 and S2; Fig. 3C), in agreement with anatomical mapping of spinothalamic tract projections in monkeys (Dum et al., 2009) and the evidence of parallel S1 and S2 activity in response to somatosensory (Klingner et al., 2015) and nociceptive stimuli in humans (Ploner et al., 1999, Liang et al., 2011, Bastuji et al., 2016). The inputs were modeled as a Gaussian function with a prior mean latency of 36 ms post-stimulus and a prior standard deviation of 16 ms. This prior latency did not overlap with the artefact period (0–20 ms). The models were furnished with a spatial forward model, mapping from the modeled source dipoles to observed MEG data, based on a single shell (Nolte, 2003).

#### Canonical microcircuit model

In the DCM analysis, each region was modeled using neural masses corresponding to different populations that comprise a canonical microcircuit (Bastos et al., 2012, Pinotsis et al., 2012). The dynamics at each region are prescribed by ordinary differential equations coupling the changes in postsynaptic voltage (V) to the changes in current (I) of each of the four neuronal populations (subscript SS: spiny stellate cells in layer 4; SP: superficial pyramidal cells in layers 2/3; DP: deep pyramidal cells in layers 5/6; II: inhibitory interneurons; Fig. 3D):

$${\dot{V}}_{\mathrm{SS}}=I_{\mathrm{SS}}$$

$${\dot{I}}_{\mathrm{SS}}=\kappa_{\mathrm{SS}}\left(A^{F}\sigma \left(V_{\mathrm{SP}}\right)-\gamma_{\mathrm{SS}\to \mathrm{SS}}\sigma \left(V_{\mathrm{SS}}\right)-\gamma_{\mathrm{SP}\to \mathrm{SS}}\sigma \left(V_{\mathrm{SP}}\right)-\gamma_{\mathrm{II}\to \mathrm{SS}}\sigma \left(V_{\mathrm{II}}\right)\mathrm{Cu}\right)-{2\kappa_{\mathrm{SS}}V}_{\mathrm{SS}}-\kappa_{\mathrm{SS}}^{2}I_{\mathrm{SS}}$$

$${\dot{V}}_{\mathrm{II}}=I_{\mathrm{II}}$$

$${\dot{I}}_{\mathrm{II}}=\kappa_{\mathrm{II}}\left(-A^{B}\sigma \left(V_{\mathrm{DP}}\right)+\gamma_{\mathrm{SS}\to \mathrm{II}}\sigma \left(V_{\mathrm{SS}}\right)+\gamma_{\mathrm{DP}\to \mathrm{II}}\sigma \left(V_{\mathrm{DP}}\right)-\gamma_{\mathrm{II}\to \mathrm{II}}\sigma \left(V_{\mathrm{II}}\right)\right)-{2\kappa_{\mathrm{II}}V}_{\mathrm{II}}-\kappa_{\mathrm{II}}^{2}I_{\mathrm{II}}$$

$${\dot{V}}_{\mathrm{SP}}=I_{\mathrm{SP}}$$

$${\dot{I}}_{\mathrm{SP}}=\kappa_{\mathrm{SP}}\left({-A}^{B}\sigma \left(V_{\mathrm{DP}}\right)+\gamma_{\mathrm{SS}\to \mathrm{SP}}\sigma \left(V_{\mathrm{SS}}\right)-\gamma_{\mathrm{SP}\to \mathrm{SP}}\sigma \left(V_{\mathrm{SP}}\right)\right)-{2\kappa_{\mathrm{SP}}V}_{\mathrm{SP}}-\kappa_{\mathrm{SP}}^{2}I_{\mathrm{SP}}$$

$${\dot{V}}_{\mathrm{DP}}=I_{\mathrm{DP}}$$

$${\dot{I}}_{\mathrm{DP}}=\kappa_{\mathrm{DP}}\left(A^{F}\sigma \left(V_{\mathrm{SP}}\right)-\gamma_{\mathrm{DP}\to \mathrm{DP}}\sigma \left(V_{\mathrm{DP}}\right)-\gamma_{\mathrm{II}\to \mathrm{DP}}\sigma \left(V_{\mathrm{II}}\right)\right)-{2\kappa_{\mathrm{DP}}V}_{\mathrm{DP}}-\kappa_{\mathrm{DP}}^{2}I_{\mathrm{DP}}$$

Here, A^F^ and A^B^ denote the ascending (i.e., forward) and descending (i.e., backward) connections between regions (i.e., extrinsic connectivity), and γ_m→n_ the connections from population *m* to *n* within regions (i.e., intrinsic connectivity). Importantly, ascending connections are excitatory and descending connections inhibitory, due to their mediation by inhibitory interneurons (Bastos et al., 2012). Similarly, all intrinsic self-connections are modeled as polysynaptically inhibiting their target populations (Auksztulewicz and Friston, 2015). The symbol σ denotes a sigmoid operator, transforming the post-synaptic potential into pre-synaptic firing rate and C scales the thalamic input u, while K symbolizes a synaptic rate constant. Given a considerable degree of realism in modelling laminar asymmetry in terms of the origin and target of ascending connections (i.e., from superficial pyramidal cells to spiny stellate cells and deep pyramidal cells) and descending connections (i.e., from deep pyramidal cells to inhibitory interneurons and superficial pyramidal cells), this model has been used in several previous studies of synaptic gain (Brown and Friston, 2013, Moran et al., 2013, Auksztulewicz and Friston, 2015).

#### Step 1: model structure optimization

In the first step, the basic structure of the model was optimized using fixed-effects Bayesian model comparison using the grand-averaged evoked responses to unattended deviants. We entertained a fairly comprehensive set of models, because the architecture underlying our somatosensory oddball paradigm has not been previously identified (in contrast to the architecture underlying mismatch negativity responses in the auditory domain). We considered a model space comprising 24 alternative network architectures. All models included 8 cortical regions (bilateral S1, S2, IFG and IPC) and differed with respect to different combinations of between-region connections (Fig. 3A). For example, the simplest model included connections between bilateral S1 and S2, while the full model included connections between somatosensory and fronto-parietal regions. When present, intra-hemispheric and inter-hemispheric connections were reciprocal; i.e., with both forward and backward extrinsic connections. Specifically, the models were partitioned based on the inclusion of IFG only (models 1–3), IPC only (models 13–15) or both IFG and IPC (models 4–12 and 16–24). When IFG and/or IPC were included, we specified inter-hemispheric connections between homotopic frontal and/or parietal areas. Different patterns of extrinsic intra-hemispheric connections between the two somatosensory and the fronto-parietal regions were tested.

In Fig. 3A, Models 1–3 included bilateral S1, S2 and IFG and connections between the somatosensory regions and the frontal source. Models 13–15 comprised bilateral S1, S2 and IPC and connections between the somatosensory regions and the parietal source. Models 4–12 and 16–24 included bilateral S1, S2, IFG and IPC, as well as different combinations of connections between the somatosensory regions and the fronto-parietal sources. Crucially, the model space was designed to test (1) whether the model evidence was significantly improved when both IFG and IPC regions were included; (2) which fronto-parietal region corresponded to the highest hierarchical level in the network; (3) whether S1 and/or S2 were effectively connected to IFG and/or IPC. Models 4–6 and 16–18 had connections between S1 and IFG, but lacked S1-IPC connectivity. Vice versa, models 7–9 and 19–21 had connections between S1 and IPC, but lacked S1-IFG connectivity. Models 10–12 and 22–24 included connections from S1 to both IFG and IPC. We reiterated the connections between S2 and the fronto-parietal regions, following the same logic. Models [4, 7, 10, 16, 19, and 22] had connections between S2 and IFG, but lacked S2-IPC connectivity. Vice versa, models [5, 8, 11, 17, 20, and 23] had connections between S2 and IPC, but lacked S2-IFG connectivity. Finally, models [6, 9, 12, 18, 21, and 24] included connections from S2 to both IFG and IPC.

#### Step 2: intrinsic and extrinsic connectivity optimization

The DCM with the highest model evidence was then optimized with respect to contextual (condition specific) modulations of intrinsic and extrinsic connections (Fig. 3B). Three contextual effects were included: (1) attended vs. unattended pain (top-down attention modulation), (2) left deviant vs. left standard (left expectation violation modulation), (3) right deviant vs. right standard (right expectation violation modulation). A set of 13 alternative models of top-down attentional modulation allowed for all combinations of changes in intrinsic gain of bilateral S1, S2, IFG, and/or IPC. To limit the model space and in agreement with previous literature (Brown and Friston, 2013), the modulation of recurrent extrinsic connectivity by top-down attention was not tested (Brown and Friston, 2013). Although endogenous attentional manipulations are clearly top-down, the attentional gain produced by these top-down effects is generally expressed at lower (sensory or domain specific) levels of cortical hierarchies.

Conversely, the models of expectation violation allowed a joint modulation of intrinsic and extrinsic connectivity by expectation violation (Garrido et al., 2007, Garrido et al., 2008, Dietz et al., 2014). With respect to intrinsic connectivity, the models allowed for all combinations of changes in intrinsic gain of S1, S2, IFG, and/or IPC (Fig. 3B, Bilateral), as well as a further distinction between contralateral or bilateral modulation of S1 and S2 gain (Fig. 3B, Contralateral/Bilateral). These included 12 alternative models of bottom-up attention modulation allowing bilateral combinations of changes in intrinsic gain of the 8 sources, as well as 16 alternative models of expectation violation modulation – allowing combinations of changes in intrinsic gain of contralateral somatosensory and bilateral fronto-parietal sources. The latter models included subsets of the following cases: contralateral S1, bilateral IFG and/or IPC (N=4); contralateral S2, bilateral IFG and/or IPC (N=4); contralateral S1 and S2, bilateral IFG and/or IPC (N=4); contralateral S1, bilateral S2, IFG and/or IPC (N=4). Finally, we specified a null model with no intrinsic modulation. With respect to extrinsic connectivity, all 29 alternative models allowed changes in recurrent (feedforward and feedback) connectivity.

Based on reconstructed source activity estimates (see below), the modulatory effect of expectation violation was modeled as an interaction with lateralization in terms of the main effect of laterality plus an ipsilateral effect of violation. In other words, expectation violation was modeled as increasing one or more connections in the hemisphere contralateral to stimulation, with an additional increase when the stimulus was unpredicted or surprising. These modulatory effects were assumed to operate on the homologous connections in each hemisphere.

The ensuing model space comprised 337 models (i.e., 13 bilateral models of top-down attention×29 bilateral/contralateral models of expectation violation). Each model was fitted to the grand-average ERF data, under the assumption that each participant had the same functional architecture. Models were compared using fixed-effects Bayesian model comparison based on the free-energy approximation to the model log-evidence (Friston et al., 2007), which embodies a trade-off between model accuracy and complexity (Penny, 2012). To accommodate uncertainty about which was the best model, we used Bayesian model averaging (Hoeting et al., 1999; Penny et al., 2010) to produce quantitative posterior estimates of effective connectivity in response to expectation violation and attention. BMA does not rely on the parameter estimates of a particular model, but instead uses the entire model space by assigning a weight to the parameters of each model according to its model evidence.

#### Step 3: between-subject variability on pain perception and DCM connectivity

As a last validation step, we asked whether inter-individual variability in the attentional modulation of subjective ratings of pain correlated with the strength of intrinsic and extrinsic connectivity. At the perceptual level, pain modulation by attention was indexed by the difference between pain ratings associated with attended and unattended blocks of painful trials. The greater this difference, the greater the attentional modulation. At the neural level, we fitted the grand-average winning model to single-subject data and tested for random (between-subject) effects on the connection strengths using a parametric empirical Bayes (PEB) procedure (Friston et al., 2016). This procedure used a between-subject GLM, with a first regressor modelling the mean connection strength across subjects and a second (Z-scored) regressor modelling attentional effects. With respect to conventional summary statistic tests, this empirical Bayesian procedure takes into account the estimate of each connectivity parameter and its estimated uncertainty. We then used Bayesian model reduction (Friston et al., 2016) to prune redundant connections that did not show a significant departure from the prior mean (of zero) or pain modulation. This enabled us to identify connections for which there was strong evidence for an effect of attentional modulation (i.e., with a posterior probability >95%). Finally, to assess the predictive validity of the surviving DCM parameters, we performed leave-one-out cross validation (Friston et al., 2016). This allowed us to quantify the ‘out of sample’ effect size in terms of the correlation between pain modulation ratings and DCM parameters. This empirical Bayesian analysis of (subject specific) connectivity estimates furnishes a predictive validity for the DCM by showing that it is possible to predict the extent of subjective pain modulation by attention using connectivity estimates based only on neurophysiological responses.

## Results

### Behavioral and subjective results

When pain was attended, participants accurately counted 95.82±1.28% of switches in spatial location from one hand to another. When pain was unattended, participants accurately counted 94.32±3.00% of switches in the color of the fixation cross. No difference was found in the detection accuracy between the two tasks; suggesting that participants were similarly engaged in both attention conditions. Further, the statistical analysis of the pain ratings indicated that participants perceived the painful stimuli as less intense when pain was unattended compared to attended, and in a similar fashion for the left and right hand (Fig. 1C). We thus found a main effect of top-down attention, *F*_(1,21)_=10.01, *p*<.005, partial *η*^2^=.32. There was no main effect of laterality or attention by laterality interaction. Finally, the difference between left and right pain ratings did not correlate with the participants’ handedness, as measured by the Edinburgh Inventory (*r*(20)=−.22, *p*=.33). In summary, these results replicated the classic findings of attentional modulation of pain (i.e., and analgesic effect of distraction), as attended painful events were consistently rated as more intense than unattended ones.

### Event-related fields and cortical generators – sensor and source space results

#### Laterality main effect

Left vs. right painful stimulation evoked increased ERF amplitudes at 53–153 ms over a right fronto-temporal region (peak-level T_max_=6.28; cluster-level p_FWE_<.001). At the source level, this effect was associated with increased responses in contralateral S1 (MNI coordinates: 32 −24 62; peak-level T_max_=4.62; peak-level p_UNC_<.001) and contralateral S2. Conversely, right vs. left painful stimulation evoked increased ERF amplitudes at 93–220 ms over a left fronto-temporal region (peak-level T_max_=7.31; cluster-level p_FWE_<.001). At the source level, this effect was associated with increased responses in contralateral S1 (MNI coordinates: −26 −36 62; peak-level T_max_=4.50; peak-level p_UNC_<.001) and contralateral S2. In summary, laterality effects were most pronounced in early-mid latency time windows and were associated with increased field strength in contralateral primary and secondary somatosensory regions.

#### Attention main effect

Attended vs. unattended painful stimuli elicited greater ERF amplitudes in both early and late time intervals, at 83–200 ms (peak-level T_max_=4.79; cluster-level p_FWE_<.001) and at 323–393 ms (peak-level T_max_=4.95; cluster-level p_FWE_<.001) over frontal, parietal, temporal and occipital sensors. At the source level, the attention effect was primarily associated with increased response of left S2 (MNI coordinates: −64 −14 18; peak-level T_max_=5.89; peak-level p_UNC_<.001) and right S2 (MNI coordinates: 62 −22 26; peak-level T_max_=4.28; peak-level p_UNC_<.001). To a lesser extent, other regions were identified in visual and primary somatosensory regions.

#### Expectation violation main effect

Deviant vs. standard painful stimuli elicited increased ERF amplitudes at 150–400 ms over most sensors (peak-level T_max_=6.25; cluster-level p_FWE_<.001), with peak responses over left fronto-temporal sensors. At the source level, the effect was associated with an extensive network of cortical sources, including bilateral primary and secondary somatosensory cortices, as well as right IPC (MNI coordinates: 36 −66 40; peak-level T_max_=4.16; peak-level p_UNC_<.001), and left IFG (MNI coordinates: −54 8 16; peak-level T_max_=3.13; peak-level p_UNC_=.001).

#### Attention by expectation violation interaction

Attended deviants and unattended standards elicited greater ERF amplitudes than unattended deviants and attended standards at 177–263 ms over a left fronto-temporal region (peak-level T_max_=3.98; cluster-level p_FWE_=.001); at 260–363 ms over a central occipital region (peak-level T_max_=4.56; cluster-level p_FWE_<.001). At the source level, this interaction was associated with an increased response of left IPC (MNI coordinates: −32 −64 46; peak-level T_max_=4.87; peak-level p_UNC_<.001) and right IFG (MNI coordinates: 50 0 10; peak-level T_max_=3.15; peak-level p_UNC_=.001).

#### Laterality by expectation violation interaction

Right deviant and left standard stimuli evoked greater ERF amplitudes than left deviant and right standard at 117–160 ms over a left fronto-temporal region (peak-level T_max_=4.19; cluster-level p_FWE_<.001). At the source level, this interaction was associated with increased responses of left S2 (peak-level T_max_=3.30; peak-level p_UNC_=.001).

### Dynamic causal modelling – connectivity results

#### Step I: model optimization

Given the selected candidate sources and their prior locations, we first optimized the model structure in a fixed-effects Bayesian model selection among 24 alternative models of grand-average responses to unattended deviants. We thus identified the model structure that best explained our data, in terms of (1) inclusion of a frontal and/or a parietal region, (2) hierarchical arrangement of the fronto-parietal sources and (3) connectivity between the somatosensory and the fronto-parietal regions. The winning model (M10) was associated with a log-evidence greater than 4.04 with respect to the second best model (M23; Fig. 3E). This corresponds to strong evidence in favor of the winning model (Penny et al., 2004). The selected model structure comprised all eight sources, with IPC modeled as hierarchically above IFG, reciprocal connections between S1 and both fronto-parietal regions and between S2 and IFG (Fig. 3A, M10). Pleasingly, Dietz et al. (2014) identified the same hierarchical organization of IPC and IFG with respect to early sensory regions during left and right stimuli in the auditory modality.

#### Step 2: intrinsic/extrinsic connectivity optimization

**Fig. 4:**
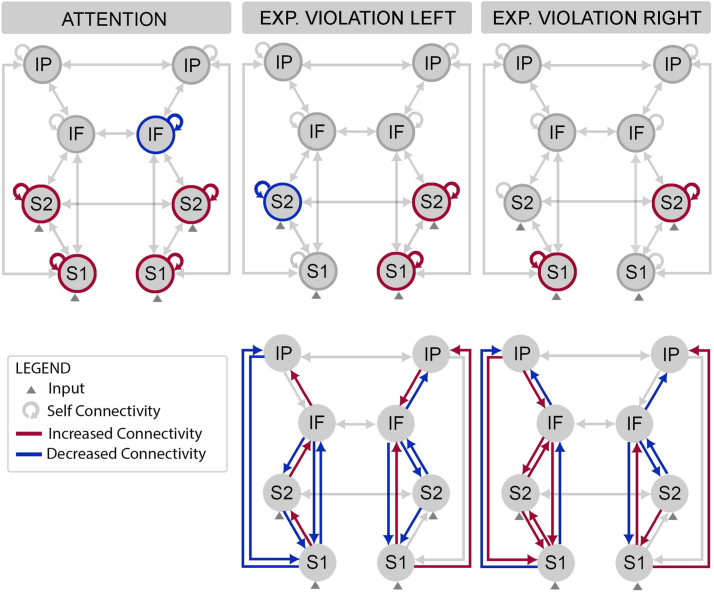
Bayesian Model Averaging (BMA) of the contextual modulation of intrinsic connectivity by attention, as well as intrinsic and extrinsic connectivity by expectation violation. Increased connectivity is showed in red, while decreased connectivity in blue. Attention increased (disinhibition) somatosensory gain, while decreasing (increased inhibition) frontal gain. Expectation violation increased the gain of contralateral primary and bilateral secondary somatosensory cortex. Further, expectation violation increased forward connectivity, in a right-lateralized fashion irrespective of violation location, while decreasing backward connectivity mostly between contralateral regions. (For interpretation of the references to color in this figure legend, the reader is referred to the web version of this article.)

The winning model was further optimized with respect to contextual changes in extrinsic and intrinsic connectivity. To this aim, we inverted models from a factorial model space, with the experimental factors (1) top-down attention, (2) left expectation violation (i.e., unexpected pain on the left hand), (3) right expectation violation (i.e., unexpected pain on the right hand) modulating a different subset of intrinsic and/or extrinsic connections. These models were fitted to grand-averaged evoked responses and the modulatory effects served to explain the observed differences in ERF amplitude. A fixed-effects Bayesian model selection suggested that the winning model allowed for (1) attentional modulation of intrinsic connectivity in bilateral SI, SII and IFG (Fig. 3B, Bilateral, M10), (2) expectation modulation of intrinsic connectivity in contralateral SI and bilateral SII (Fig. 3B, Contralateral/Bilateral, M13), and (3) expectation modulation of recurrent extrinsic connectivity throughout the hierarchy. The difference in log-evidence compared to the second-best model was 190.97 (Fig. 3E, B Matrix), corresponding to a very strong evidence for the winning model (i.e., posterior probability>99%). The winning model provided an excellent fit between predicted and observed data for all sensors and time points used for model inversion. However, to account for residual uncertainty about the best model, Bayesian model averaging (BMA) was used to provide the quantitative estimates of effective connectivity and their modulation by contextual factors (Fig. 4).

Following BMA, significant changes in effective connectivity corresponded to a posterior probability>99%. The gain of superficial pyramidal cells in bilateral S1 and S2 was increased under attention, reflecting a significant decrease in self-inhibition (i.e., disinhibition or increased gain). In contrast, the gain of superficial pyramidal cells in rIFG was decreased under attention, while it did not change markedly in lIFG. Furthermore, the gain of superficial pyramidal cells in contralateral S1 increased following expectation violation, reflecting a significant decrease in self-inhibition in S1 contralateral to stimulus presentation. In S2, left deviants were associated with significant disinhibition in the contralateral hemisphere and inhibition in the ipsilateral hemisphere, while right deviants lead to a nominal disinhibition in the contralateral hemisphere and a significant disinhibition in the ipsilateral hemisphere. With respect to extrinsic forward connectivity, our findings showed lateralized changes in forward connectivity, independent of the expected location, with an increased influence from rS1 to right higher-level regions, but decreased influence from lS1 to left higher-level regions. In a similarly lateralized fashion, effective connectivity from S1 to S2 to IFG to IPC was increased in the left hemisphere, but decreased in the right hemisphere.

The descending connectivity modulation showed an asymmetric pattern, with left deviants associated with increased bilateral inhibition of S1 by descending connections from S2, and right deviants associated with decreased bilateral inhibition of S1 from S2. In addition, left deviants were associated with increased bilateral downstream inhibition with the exception of disinhibition of rIPC influences on rIFG. In contrast, right deviants were associated with increased left-hemisphere disinhibition at all hierarchical levels, and an overall increased right-hemisphere inhibition (Fig. 4). These results suggest that location-based expectation violation is mediated by location-independent changes in forward connectivity. However, it involves lateralized changes in backward connectivity that specifically depend on the unexpected location.

#### Step 3: between-subject variability on pain perception and DCM parameters

**Fig. 5:**
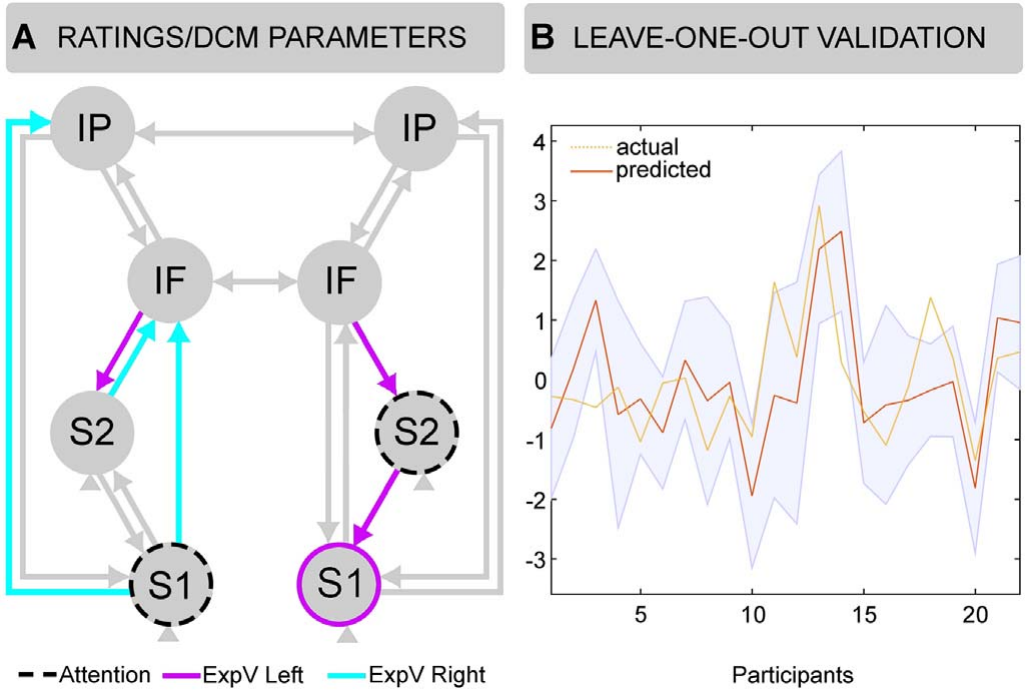
**A)** Parametric empirical Bayes analysis revealed that inter-individual variability in pain modulation ratings correlated with intrinsic and extrinsic connectivity changes driven by attention and expectation violation. The neural effect of attention on self-inhibitory connections in left S1 and right S2 correlated with the degree to which participants experienced pain enhancement by attention. Further, following left expectation violation, pain modulation ratings were predicted by the strength of backward connectivity to somatosensory regions primarily in the right hemisphere. Instead, following right expectation violation, pain modulation ratings were predicted by the strength of forwards connectivity from S1 and S2 to higher order regions in the left hemisphere. This pattern of results complemented hemispheric functional asymmetries observed at the group level. **B)** Prediction accuracy at the leave-one-out cross validation, which allowed us to quantify the ‘out of sample’ effect size in terms of the correlation between pain modulation ratings and DCM parameters. The analysis revealed a subset of intrinsic and extrinsic parameters that successfully predicted pain modulation ratings (*r*(20)=.55, *p*<.01). The x-axis represents single participants (N=22), while the y-axis depicts the actual vs. predicted z-scored pain modulation ratings.

We found that subjective ratings of pain modulation by attention can be predicted (over subjects) by the degree of self-inhibition in left S1 and right S2 under attention. Further, following left expectation violations, pain modulation ratings predicted the strength of self-inhibition in right S1 and backward connectivity from right IFG to right S2 to right S1, as well as from left IFG to left S2. On the other hand, following right expectation violation, pain modulation ratings predicted the strength of forward connectivity from left S1 and S2 to higher-order regions (Fig. 5). The laterality of DCM parameters covarying with perceptual modulation ratings was consistent with the contralateral projections of incoming sensory signals. However, the asymmetry in right hemisphere backward connectivity vs. left hemisphere forward connectivity complemented functional differences observed in response to expectation violation at the grand-average level.

Following the leave-one-out procedure, our analysis revealed that the subset of identified parameters successfully predicted pain modulation ratings (*r*(20)=.55, *p*<.01). These results are consistent with the notion that pain perception is not strictly related to any single area or connection, but with patterns of intrinsic and extrinsic connectivity modulation throughout the somatosensory hierarchy. Note that this (non-trivial) effect size (corresponding to over 25% variance explained) was based upon cross-validation and is therefore an out-of-sample estimate. In other words, this is the sort of predictability one would expect by performing DCM on a new subject to estimate their propensity for pain modulation by attention.

## Discussion

We used magnetoencephalography (MEG) and dynamic causal modelling (DCM) to investigate how expectancy and attention processes influence effective connectivity in a somatosensory network, in the context of pain perception. Overall, both expectation violation and attention to pain influenced the promulgation of painful somatosensory inputs throughout a hierarchy of brain sources; including primary and secondary somatosensory cortex (S1 and S2), inferior frontal gyrus (IFG) and inferior parietal cortex (IPC). Crucially, we showed that a similar gain control mechanism governs the influence of expectancy and attentional effects on pain-related neural processing. This mechanism is consistent with an enhanced precision-weighting of prediction errors in S1 and S2. Expectation violation also modulated forward and backward connections between somatosensory and fronto-parietal sources. These findings generalize the interpretation of mismatch responses as prediction error minimization and adjustment of a predictive model of sensory causes (Garrido et al., 2009; Lieder et al., 2013) to the domain of painful stimuli. Interestingly, expectation violation increased forward connectivity in a similar fashion for left and right deviants, while inducing mostly contralateral changes in backward connectivity, depending on where the unexpected stimulus occurred. Finally, we revealed that intrinsic and extrinsic connectivity DCM parameters jointly predicted subjective pain modulation ratings.

### The somatosensory hierarchy underlying the spatial localization of pain

We found that the network architecture best accounting for responses during spatial location of painful events produced neural responses in contralateral S1 and bilateral S2, as well as IFG and IPC. Specifically, the winning architecture implicated bidirectional connections from somatosensory regions to IFG and IPC, with the parietal node at the highest hierarchical level. This hierarchical architecture is in agreement with recent findings in the auditory system (Dietz et al., 2014) and may be mediated anatomically by the third branch of the superior longitudinal fasciculus (Thiebaut de Schotten et al., 2011).

Specifically, at lower hierarchical levels, sensory inputs from the skin are conveyed by mechanoreceptive and nociceptive afferents projecting to spatially organized somatotopic maps at spinal, thalamic and cortical levels of the somatosensory system. At higher order levels, fine-grained somatotopic maps of tactile and nociceptive inputs are encoded in contralateral primary S1 (Mancini et al., 2012) and, with lessened precision, in S2 and insular cortex (Brooks et al., 2005, Mazzola et al., 2009, Baumgärtner et al., 2010). Topographic organization is commonly viewed as an integral part of a neuronal encoding that underlies localization ability; however, compelling evidence supports the notion that activity within these brain regions is not sufficient for the perception of spatial attributes of somatosensory experience. In particular, the detection and localization of painful events have been associated with the recruitment of an extensive network of brain regions beyond somatosensory areas and including fronto-parietal regions (Oshiro et al., 2007, Oshiro et al., 2009).

### Both expectation violation and attention to pain modulate intrinsic connectivity in somatosensory cortex

Both unexpected and attended pain influenced the gain of S1 and S2. Specifically, location-based expectation violation elicited a redeployment of precision between left and right S1, by disinhibiting contralateral but not ipsilateral superficial pyramidal cells in primary regions. Further, expectation violation also influenced gain control in S2, but in a lateralized fashion. These findings suggest that spatial expectation violation selectively enhanced cortical gain in contralateral S1 and right S2. While top-down attention also influenced the gain or precision of superficial pyramidal neurons in S1 and S2, its disinhibitory effects were symmetrical, in accordance with the non-spatial top-down attention manipulation. Further, attention to pain was also associated with increased inhibition of right IFG.

From a general perspective, a recent theory of attention – within the predictive coding framework – suggests that local changes in cortical gain are a key mechanism in both top-down attention and bottom-up salience (Feldman and Friston, 2010). Neurophysiologically, gain corresponds to the sensitivity of neurons responding to prediction errors (i.e., superficial pyramidal cells), resulting from the competition between excitatory and inhibitory neuronal populations. Functionally, increased somatosensory gain would lead to the increased influence of low-level sensory responses on higher-level processing in frontal and parietal regions observed here. Furthermore, the implicit decreased precision-weighting of right IFG signals by attention, is consistent with enhanced weighting of low-level somatosensory signals. This interpretation is supported by a previous EEG study demonstrating that tactile mismatch signals originating in S1 and S2 encode a perceptual mechanism described as Bayesian surprise, an information theoretic index reflecting the amount of prediction error (Ostwald et al., 2012). At later time points, mismatch signals from frontal and cingulate cortex were instead associated with stimulus change or salience (Ostwald et al., 2012), likely reflecting active inferential processes. Future studies could manipulate surprise and salience orthogonally to investigate whether gain-dependent mechanisms explain not only mismatch (perception-related) signals from lower sensory regions, but also salience (action-related) signals from frontal regions.

Our finding seems at odds with previous studies reporting that the effect of attention lies in the modulation of between-region backward connectivity (Auksztulewicz and Friston, 2015, Chennu et al., 2016). However, changes in between-region backward connectivity and within-region postsynaptic gain have aligned functional interpretations. Backward connectivity refers to the modulatory effect of predictions on lower-level activity. On the other hand, postsynaptic gain reflects the precision of prediction errors, which is dependent to the degree of mismatch between prior expectations (i.e., backward connectivity) and incoming signal.

In summary, our findings showed a common precision-weighting synaptic mechanism for bottom-up and top-down attentional modulation in the somatosensory hierarchy. Importantly, these results extend previous modelling work on visual spatial attention (Brown and Friston, 2013) and auditory temporal attention (Auksztulewicz and Friston, 2015), pointing to a general predictive coding gain control mechanism, across expectancy and attentional manipulations, that may be generic to all sensory modalities.

### Expectation violation modulates extrinsic connectivity in the somatosensory hierarchy

Beyond altering intrinsic connectivity, expectation violation also increased recurrent extrinsic connectivity between somatosensory, frontal and parietal regions. This is in line with the role of gain in regulating neuronal message passing across the cortical hierarchy (Friston, 2005). We observed bilateral increases of effective connectivity driven by expectation violation, regardless the location of deviant pain. However, the pattern of ascending connectivity was asymmetrical in the two hemispheres. At each level of the neural hierarchy, incoming inputs are compared with top-down predictions, with any resulting discrepancy (i.e., error) being passed to higher regions of the hierarchy (Friston, 2008). Prediction errors thus signal the degree to which higher-level expectations about sensory causes must be revised to reduce overall surprise or free energy (Friston and Kiebel, 2009, Friston, 2010). For instance, recurrent connectivity amongst a fronto-temporo-parietal network is commonly modulated in response to a novel and unexpected change in the environment, as in the case of auditory mismatch negativity (MMN) responses (Dietz et al., 2014, Phillips et al., 2015). Further, the MNN has been shown to reflect a failure in suppressing prediction errors (Garrido et al., 2007, Garrido et al., 2008, 2009) and the adjustment (learning) of a new probabilistic model of the environment (Lieder et al., 2013a, Lieder et al., 2013b). We thus interpreted the increased bilateral (but asymmetrical) forward and backward connectivity as reflecting location-unspecific detection of expectation violation and location-specific update mechanism, corresponding to feedforward propagation of highly-precise sensory prediction errors and backward signaling of updated spatial predictions. Further, the asymmetrical forward-connectivity results are consistent with the notion of a supramodal right-lateralized fronto-parietal network that has been implicated in the reorienting of spatial attention. For instance, the bottom-up reorienting of visuo-spatial attention towards a novel location is thought to be specifically mediated by a right-lateralized ventral attention network (Corbetta and Shulman, 2002), regardless whether attention is re-oriented towards the left or the right hemi-field (Shulman et al., 2010). Finally, an alternative explanation is offered by functional asymmetries in the central representation of sympathetic and parasympathetic afferent projections (Craig, 2005). The right hemisphere is primarily associated with sympathetic activity, mediating arousal, negative affect, pain, and interoceptive processing. It is thus possible that this lateralization reflects the operation of a system generally involved in interoceptive active inference (Seth, 2013).

### Pain perception as an inferential process

In the present study, we replicated the well-known effect of attentional modulation of pain perception, as participants consistently reported less pain when noxious stimuli were unattended (e.g., Bushnell et al., 1985; Miron et al., 1989). Further, we demonstrated that the influence of attention on pain was associated with the degree to which pain-related neural signals changed intrinsic connectivity in somatosensory regions, and their feedforward and feedback message passing from and to higher-order regions. Predictive coding theories describe the brain as using probabilistic internal models to infer the causes of sensory inputs (Rao and Ballard, 1999, Friston, 2005, Clark, 2013). Within this framework, both expectation violation and attention are integral part of perceptual inference and act via precision-weighting mechanisms in order to produce context-sensitive responses (Friston, 2009). This theory fits comfortably with the evidence that perceived pain intensity is strongly regulated by top-down processes; rather than solely reflecting nociceptive bottom-up sensory inputs (for a review, see Büchel et al., 2014). Our results are consistent with a previous study showing that increased recurrent connectivity between S1 and S2 was associated with awareness of peri-threshold tactile stimuli (Auksztulewicz et al., 2012), thus indicating a link between conscious perception and the instantiation and resolution of prediction errors in somatosensory regions (also see, Auksztulewicz and Friston, 2016).

### Limitations and future directions

One limitation of the present study entails the lack of a behavioral measure of expectation violation, thus precluding an assessment of the effect of expectation on pain perception and pain-related neural measures. Furthermore, our results are limited to spatial expectation violation. An important issue for future research will be to test the association between expectations, prediction error signals and pain perception; for example, by using a cue-based paradigm combined with trial-by-trial pain ratings. Finally, it would be important to assess whether these results can be generalized to temporal and magnitude expectations (e.g., manipulation of the expected intensity, as for example in Fardo et al., 2015).

## Conclusions

Predicting coding accounts of brain function offer a unifying and principled explanation for expectation violation and attention in shaping neural responses across sensory modalities. Here, we extended previous work on the functional anatomy of mismatch responses and attention to the somatosensory domain. Our work suggests that both expectation violation and attention to pain jointly influence the cortical gain of superficial pyramidal cells in the somatosensory hierarchy. These findings shed new light on the neurobiological mechanisms associated with expectancy and attention in cortical pain-related processing.

## Conflict of interest

The authors declare no competing financial interests.

## Author contributions

F.F., M.A., M.J.D. and A.R. designed the experiment. F.F. and M.A. acquired the data. F.F., R.A. and K.J.F. analyzed the data. F.F., R.A., M.A., M.J.D., and K.J.F. wrote the paper.

## Footnotes

1 Technically, predictive coding is a particular form of Bayesian filtering; e.g., Kalman filtering. In this context, the precision corresponds to the Kalman gain. The Kalman gain is applied to prediction errors to provide a Bayes optimal update to the prediction based upon previous estimates. In other words, the precision that we are talking about here is the precision of sensory information (or ascending prediction errors at each level of the hierarchy). For this reason, we associate the encoding of uncertainty in predictive coding (in the brain) with the precision of prediction errors – supposedly encoded by superficial pyramidal cells. This means that a precise prior belief is encoded by the gain of superficial pyramidal cells high in the hierarchy that therefore exert more influence over the deep pyramidal cells (encoding expectations) at that level, relative to ascending prediction errors that convey relatively imprecise information. In short, we can interpret the excitability or postsynaptic gain of superficial pyramidal cells as mediating the precision at the level of the hierarchy occupied by those cells.
